# Longitudinal development of visual working memory precision in childhood and early adolescence

**DOI:** 10.1016/j.cogdev.2016.03.004

**Published:** 2016

**Authors:** S. Burnett Heyes, N. Zokaei, M. Husain

**Affiliations:** a School of Psychology, University of Birmingham, United Kingdom; b Department of Experimental Psychology, University of Oxford, United Kingdom; c Department of Psychiatry, University of Oxford, United Kingdom

**Keywords:** Working memory, Precision, Development, Adolescence, Childhood

## Abstract

Visual working memory (VWM) is the ability to hold in mind visual information for brief periods of time. The current study investigated VWM precision development longitudinally. Participants (N = 40, aged 7–11 years) completed delayed reproduction sequential VWM tasks at baseline and two years later. Results show age-related improvement in recall precision on both 1-item and 3-item VWM tasks, suggesting development during childhood and early adolescence in the resolution with which both single and multiple items are stored in VWM. Probabilistic modelling of response distribution data suggests age-related improvement in precision is attributable to a specific decrease in the variability (noisiness) of stored feature representations. This highlights a novel developmental mechanism which may underlie longitudinal improvement in VWM performance, crucially without invoking improvement in the number of items that can be stored. VWM precision provides a sensitive metric with which to track developmental changes longitudinally, shedding light on underlying cognitive mechanisms.

## Highlights

• Longitudinal development of visual working memory (VWM) was assessed in children.
• Recall precision rather than span or item capacity was measured.
• VWM precision increased significantly over two years.
• Modelling revealed this was due to decreased variability (noise) in VWM.
• Improvement in VWM recall with development occurs even for a single retained item.

## 1 Introduction

Visual working memory (VWM) provides a temporary storage mechanism for the retention and manipulation of visual information to support other cognitive processes (Luck & Vogel, 1997). This ability is considered to be a critical contributor to many essential cognitive functions such as decision-making, complex reasoning and goal-directed action (Baddeley, 2003). Numerous developmental studies have shown that performance on established neuropsychological tests of VWM improves during childhood (Alloway, Gathercole, & Pickering, 2006; Gathercole, Pickering, Ambridge, & Wearing, 2004). Typically in these tests, participants view a static visual array (e.g. coloured shapes) or spatiotemporal sequence of visual events (e.g. block tapping) which are held in memory and then reproduced following a delay. These studies have demonstrated, within large cross-sectional datasets, robust age trajectories and evidence for developmental stability in the relationship of VWM to other cognitive components (Gathercole et al., 2004).

The mechanisms underlying VWM development and its relationship with other cognitive measures remain debated (Astle & Scerif, 2011). That is, what are the fundamental cognitive mechanisms underlying developmental improvements in VWM? Most traditional measures of VWM rely on indices of the *number of items* that can be remembered, e.g. using tasks that measure recall span or change detection. These reports rely on the classical concept that VWM is limited to holding only a very small number of items, as little as 3 or 4 in adults, arguing in favour of a ‘quantal’ memory mechanism limited to 3–4 memory ‘slots’(Luck & Vogel, 1997). In effect, this is a digital view of how items are stored in memory, with both span and change detection tasks providing a discrete estimate of the number of items that can be retained in VWM.

Recently, an innovative empirical approach has shown potential to accurately and sensitively characterise VWM performance in a completely different way. Unlike span or change detection tasks, this approach relies on participants reproducing the exact qualities of the retained information on a continuous response scale, e.g. the orientation of a bar stimulus. Such analogue responses provide a measure of working memory *precision*, where precision reflects the resolution with which items are held in VWM (Bays, Catalao, & Husain, 2009; Fougnie, Asplund, & Marois, 2010; Wilken and Ma, 2004, Zhang and Luck, 2008). Recent investigations suggest that continuous recall measures may be more sensitive indices of changes in VWM than discrete capacity measures, e.g. in adult neuropsychological populations (Zokaei, Burnett Heyes, Gorgoraptis, Budhdeo, & Husain, 2014).

Findings from such delayed reproduction tasks have also challenged the ‘quantal’ view of VWM. Results from such studies are not consistent with the view that there is a fixed upper limit to the number of items that can be stored in VWM (Bays et al., 2009; Gorgoraptis, Catalao, Bays, & Husain, 2011; Ma, Husain, & Bays, 2014; Zokaei, Gorgoraptis, Bahrami, Bays, & Husain, 2011). Instead, these investigations have shown that response data can be modelled by considering VWM to consist of a limited cognitive resource (Bays & Husain, 2008), with recall error arising due to noise in tuned populations of neurons (Bays, 2014, Bays, 2015, Ma et al., 2014). As the number of items stored increases, the precision with which each item can be retained decreases, purportedly because the same pool of tuned neurons must represent more items, and thus the representation of each item becomes noisier (Bays, 2014, Bays, 2015, Ma et al., 2014).

Using the statistical procedure of mixture modelling of response data, continuous VWM tasks also provide a means to dissect out *sources of error* contributing to the pattern of overall VWM performance (Bays et al., 2009). In other words, what basic, fundamental cognitive processes contribute to working memory failure and success? Firstly, response error can theoretically arise due to variability in memory – ‘noise’ – associated with the remembered feature; that is, how accurately information (e.g. orientation) is stored. Alternatively, error can arise due to random guessing, for example, due to failures at encoding or at retrieval leading to a completely random response. Lastly, error may arise due to systematic interference or corruption of information by the other items encoded into VWM. These latter *misbinding responses* occur when one feature of an object (e.g. colour) is erroneously linked with the feature (e.g. orientation) of a different object stored in memory (Bays et al., 2009). Crucially, characterising VWM development in terms of these distinct sources of error may provide further insights into underlying developmental cognitive mechanisms.

The current study investigated development of VWM precision longitudinally between age 7 and 13 years. Forty participants completed an identical task battery twice, two years apart. Tasks consisted of 1-item and 3-item sequential VWM precision tasks and a sensorimotor control task. Precision was calculated as 1/SD of error (reciprocal of variability) in response. After controlling for developmental changes in sensorimotor performance, we predicted longitudinal increases in recall precision on both the 1-item and 3-item VWM tasks. In addition, we predicted mixture modelling of recall data from the 3-item VWM task would provide evidence that effects of age are attributable specifically to a decrease in variability in the representation of target stimuli, and not to any changes in random guessing or misbinding. That is, the mixture modelling would shed further light on more fundamental cognitive mechanisms underlying memory development—whether changes in VWM performance are due to developmental increases in continuous memory resource, or to some other process such as reduced frequency of misbinding or guessing.

## 2 Methods

### 2.1 Participants

40 participants were recruited from a single-sex (male) preparatory school in Oxfordshire and tested on the same protocol twice, two years apart (*t*1 and *t*2). Participant age at *t*1 ranged from 7.9 to 11.7 years, with mean 10.2 and standard deviation (SD) 1.02 (see Table 1). Participants were from a larger (*N* = 90) cross-sectional cohort (Burnett Heyes, Zokaei, van der Staaij, Bays, & Husain, 2012). The current longitudinal cohort of 40 participants consisted of all *t*1 participants who were still attending the school at *t*2 for whom parent/guardian consent could be obtained at *t*2 and for whom timetabling constraints permitted attendance of the testing session at *t*2.

### 2.2 FSIQ^e^

Standardized yearly test scores (CAT-3; www.gl-assessment.co.uk) were provided by the school for each participant at *t*1 and transformed into estimated full-scale IQ (FSIQ^e^; see Table 1), as per Wright, Strand and Wonders (2005):

FSIQ^e^ = 1.1 × CAT-Av − 12.0

where FSIQ^e^ is estimated FSIQ and CAT-Av is average CAT-3 score calculated by combining standardised scores on verbal, non-verbal and quantitative reasoning subtests (Wright et al., 2005).

### 2.3 Tasks

#### 2.3.1 Colour-naming task

At the start of the experiment, each participant completed a colour-naming task in which they were shown five screenshots from the VWM task, each containing a bar in one of the following stimulus colours: red, yellow, green, blue and purple/pink. Participants were asked to name aloud each of these five colours. All participants successfully completed this task.

#### 2.3.2 Sensorimotor control task

**Fig. 1:**
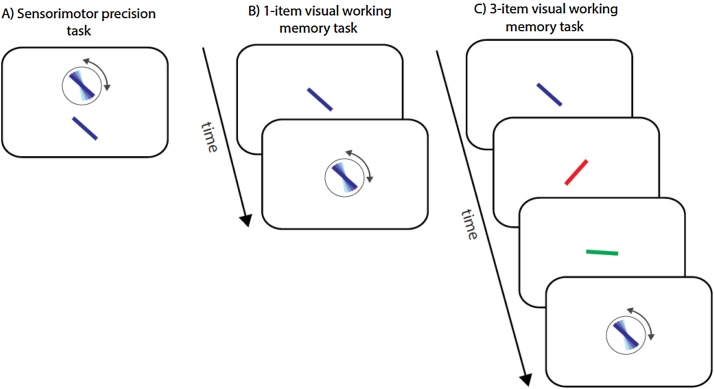
Schematic presentation of tasks. (A) Sensorimotor control task. Participants used a dial to adjust the orientation of the probe bar (above, in circle) to that of the target bar (below). (B) 1-item visual working memory task. Participants had to keep in mind the orientation of the target bar, and following a delay, used a dial to adjust the orientation of the probe bar (in circle) to that of the target bar held in memory. (C) 3-item visual working memory task. Participants were presented with a sequence of 3 coloured bars. Following a delay, a probe bar appeared (in circle). Participants used the dial to adjust the orientation of the probe bar to match the bar of the same colour from the previous sequence.

Directly after completion of the colour-naming task, participants completed 25 trials of a sensorimotor control task (Fig. 1A). Stimuli were presented on a laptop monitor (32° × 19°) at a viewing distance of ∼52 cm.

On each trial a coloured oriented bar (2 × 0.2° of visual angle) was presented against a grey background. After 500 ms following the presentation of the bar, a probe bar of the same colour surrounded by a black circle appeared below the target. Participants were asked to adjust the orientation of the probe bar using a rotating dial to match the orientation of the target which remained on screen until response. The black circle surrounding the probe item disappeared upon rotating the dial.

Bar colour was selected so as to be easily distinguishable—as in the colour-naming task. The orientation of the target and probe were independently randomized. The inter-trial interval (ITI) was 500 ms.

#### 2.3.3 Visual working memory task: 1-item

Directly after completion of the sensorimotor control task, participants completed 30 trials of a 1-item VWM task. On each trial, participants were presented with a single coloured oriented bar at the centre of the screen for 500 ms. Following a blank 500 ms delay, a probe bar of the same colour appeared at the centre of the screen surrounded by a black circle. Participants were asked to adjust the probe bar’s orientation to match the orientation of the probed (‘target’) bar using the dial (Fig. 1B).

Participants completed 30 trials of this task divided into 2 blocks of 15 trials each with a short break in between blocks. During the break participants were encouraged to focus on a far point in the room, to minimise ocular fatigue. The ITI was 500 ms.

#### 2.3.4 Visual working memory task: 3-items

Directly after completion of the 1-item VWM task, participants completed 90 trials of a 3-item VWM task. A schematic presentation of this task is shown in Fig. 1C. On each trial, a sequence of 3 coloured bars was presented at the centre of the screen against a grey background. Each bar was presented for 500 ms followed by a 500 ms blank interval prior to the presentation of the next bar. At the end of each trial, a randomly oriented probe bar of the same colour as one of the bars in the previous sequence was presented within a black circle. Using the dial, participants were asked to match the probe’s orientation to the orientation of the similarly-coloured bar in the preceding sequence (the ‘target’). All colours/serial positions in the sequence were probed with equal probability. Participants completed 90 trials of this task, with a break every 15 trials. The ITI was 500 ms.

### 2.4 Analysis

#### 2.4.1 Developmental changes in precision

We calculated recall precision as the reciprocal of the circular standard deviation of response error (where response error is the difference between response and target angles) (Fisher, 1993). Precision is a measure of response variability: less variability corresponds to more precise recall. Recall precision was calculated for the sensorimotor, 1-item and 3-item VWM tasks. To evaluate the effect of age on VWM precision controlling for any changes in sensorimotor performance, we corrected performance on the VWM task by subtracting sensorimotor error from VWM recall error and recalculating precision accordingly (i.e. 1/sqrt(difference in error variance)).

To evaluate developmental changes in precision, Wilcoxon signed-rank tests (the non-parametric equivalent of paired samples *t*-tests) were conducted comparing *t*1 vs. *t*2 corrected 1- and 3-item VWM precision values (overall and at each serial position) as well as sensorimotor precision. Statistical significance was *p* < 0.05. Non-parametric statistics were used since in our data precision is not normally distributed. Finally, to investigate differential trajectories of development for 3-item relative to 1-item VWM tasks, Wilcoxon signed-rank tests were conducted comparing *t*1 vs. *t*2 3-item VWM precision values, corrected for 1-item VWM performance by subtracting 1-item VWM recall error from overall 3-item VWM recall error and recalculating precision accordingly (i.e. 1/sqrt(difference in error variance)).

#### 2.4.2 Mixture modelling of error in response

In order to identify mechanisms underlying developmental changes in precision on the 3-item VWM task, we fit a probabilistic model that dissociates different sources of error in memory previously introduced by Bays et al. (2009) (see also http://www.sobell.ion.ucl.ac.uk/pbays/code/JV10/). This model is described by the following equation:

$$p\left(\hat{\theta }\right)=\alpha \phi_{\kappa }\left(\hat{\theta }-\theta \right)+\beta \frac{1}{\text{m}}\overset{m}{\underset{i}{\sum }}\phi_{\kappa }\left(\hat{\theta }-\phi_{i}\right)+\gamma \frac{1}{2\pi }$$

θ is the true orientation of the target item, $\hat{\theta }$ is the orientation reported by the participant and $\phi_{\kappa }$ is the von Mises distribution (the circular analogue of the Gaussian distribution) with mean zero and concentration parameter κ (kappa). The probability of reporting the target item is given by α.

**Fig. 2:**
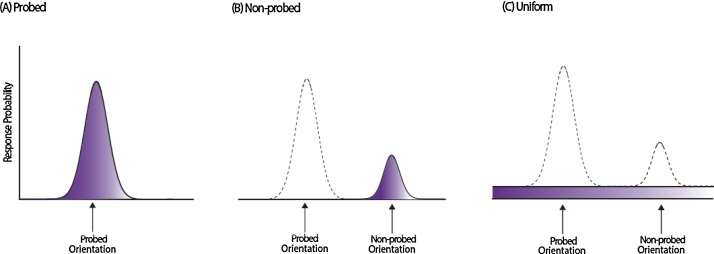
Three sources of error in memory used for modelling performance. (A) A Von Mises (circular Gaussian) distribution with concentration parameter κ, centred on the *probed* or *target* value, capturing variability in memory for the probed orientation, with the area under the distribution (shaded) being proportional to the probability of responding to the target. (B) Von Mises distribution with concentration parameter κ, centred on one of the *non-probed* or *non-target* values, resulting from errors in misidentifying the orientation which belonged to the probed colour (*misbinding*). The area under the distribution corresponds to the proportion of non-target responses. (C) Uniform distribution of error corresponding to *random error*, with the area under this distribution corresponding to the proportion of random responses.

Several sources of error could contribute to developmental changes in performance on VWM tasks such as those employed in the present study. Firstly, changes in VWM performance could result from a change in variability in memory for target features – here orientation – captured by the model concentration parameter (κ). κ is a measure of variability, where higher κ corresponds to lower variability in memory representations (Fig. 2A). Successful performance of the 3-item VWM task also requires memory for the correct *combination* of orientation and colour. Therefore, changes in performance on the 3-item VWM task could arise as a result of changes in the proportion of responses arising as a result of an incorrect conjunction of colour and orientation (misbinding errors). In such trials participants make an error centred on the orientation of other (non-probed) items in the sequence (Fig. 2B). In clearer terms: if the probed item is red but participants respond with the orientation of one of the other coloured bars in the sequence, this would be classified by the model as a misbinding error. In the model, the probability of reporting a non-target item is given by β, with {ϕ_1_, ϕ_2_ … ϕ_m_} the orientations of the *m* non-target items. Alternatively, change in recall precision could occur due to changes in the number of guesses/random responses—captured in the model by γ (Fig. 2C), where γ=1-α-β.

Maximum likelihood parameters of κ, α, β and γ were obtained for each task using an expectation maximization procedure for each participant (Myung, 2003). Using this probabilistic model, we were able to determine the underlying sources of developmental change in VWM. Similar to our analysis of recall precision, we used paired *t*-tests to compare *t*1 vs. *t*2 measures of κ and the proportion of target, non-target and random responses.

## 3 Results

### 3.1 Sensorimotor precision during development

Sensorimotor precision improved significantly between *t*1 and *t*2 (*Z* = 3.19, *p* *=* 0.01; Table 2). Therefore, in the remaining analyses of performance in the 1-item and 3-items VWM tasks, we corrected performance for changes in sensorimotor precision (for details, see Section 3).

### 3.2 Working memory precision improves with age on the 1- and 3-item VWM tasks

Mean precision on the 1-item VWM task improved significantly after 2 years after correcting for developmental change in sensorimotor precision (*Z* = 2.87, *p* = 0.004; one outlier >2.5 SD > mean excluded). Thus, precision of recall for even a single item maintained in memory increased after 2 years in childhood and early adolescence.

**Fig. 3:**
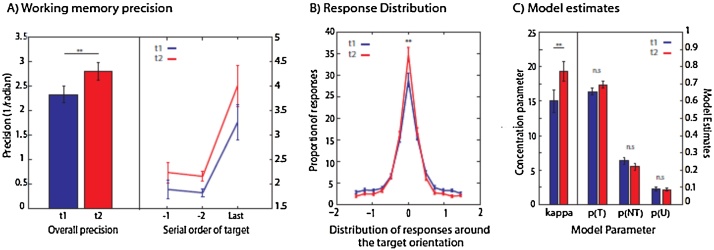
Performance on 3-item visual working memory task. (A) Mean recall precision improved over two years overall and at different serial positions. (B) Distribution of responses with respect to the orientation of the target stimuli narrows with increasing age. After two years, there was a decrease in variability in response around the target orientation. (C) Model estimates demonstrate that with age there is a change only in the concentration parameter, κ, i.e., variability in memory for target orientation.

Recall precision on the 3-item VWM task also improved significantly with age. Participants performed significantly better at *t*2 compared to *t*1 (Z = 2.39, *p* = 0.017; Fig. 3A). Wilcoxon signed-rank tests comparing *t*1 vs. *t*2 corrected precision at each serial position of the target demonstrated a significant improvement in WM precision for items presented first and second in the sequence (*Z* = 2.05, *p* = 0.04 and *Z* = 3.06, *p* = 0.002 respectively) with no significant difference for the third item (*Z* = 1.57 *p* = 0.12; Fig. 3A).

### 3.3 Improvement on 3-item WM task greater than improvement on 1-item VWM task

**Fig. 4:**
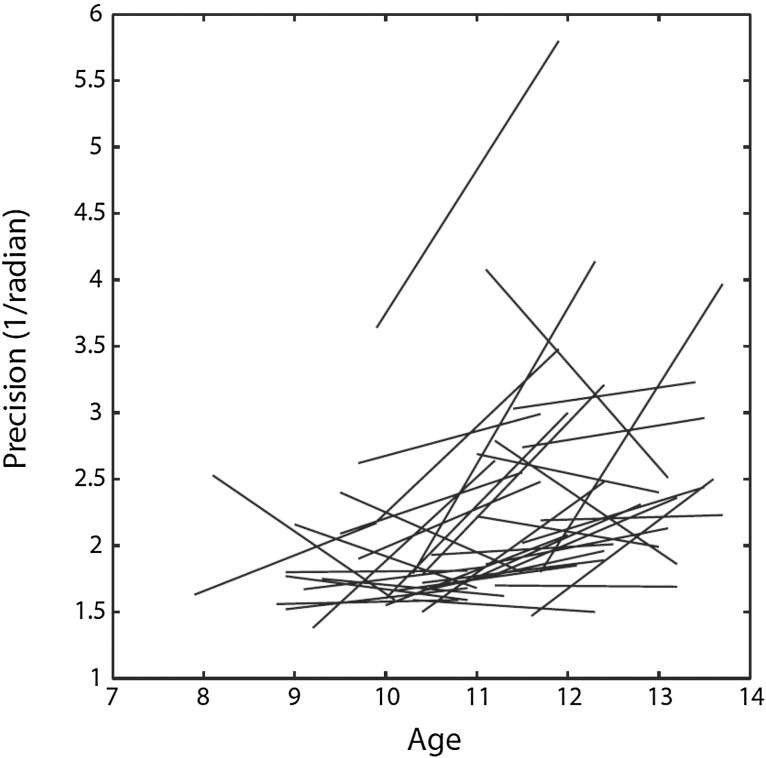
Performance on the 3-item visual working memory task for each participant at *t*1 and *t*2. Trajectory of change, corrected for change in performance on the 1-item visual working memory task.

Comparison of *t*1 vs. *t*2 3-item VWM precision corrected for 1-item VWM precision showed evidence for a steeper developmental trajectory of precision in the 3-item VWM task relative to the 1-item VWM task (*Z* = 2.79, *p* = 0.005; see Fig. 4 for individual participants’ change in precision).

### 3.4 Mixture modelling of error in response

To visualize the distribution of responses in the 3-item VWM task, we plotted the distribution of responses around the target (i.e. probed) orientation. As shown in Fig. 3B, the proportion of responses falling close to the target orientation increased from *t*1 to *t*2. This is specifically illustrated in the peak of response distribution around zero, i.e. the greater proportion of responses at the target orientation and lower proportion of responses at the tail of the distribution.

However, overall performance does not inform us as of the *sources of error*, and how these alter with age. That is, overall performance does not shed light on whether improved VWM performance is due to decreased variability in response for the target feature (kappa), changes in proportion of target (p(T)) or non-target responses (p(NT)), or increased random responses (p(U)). We therefore fit a probabilistic model (Fig. 2; Bays et al., 2009) to each participant’s 3-item VWM dataset at each time point to examine the effect of age on each of the possible sources of error.

Results show that kappa (inverse of variability in response around probed or target orientation) increased significantly with age (*t*(39) = 3.3, *p* = 0.002), crucially with no change in other model parameters: *t*(39) = 1.6, *p* = 0.12 for p(T), *t*(39) = 1.4, *p* = 0.18 for p(NT and *t*(39) = 0.3, *p* = 0.73 for p(U) (Fig. 3C). Thus variability around the probed target orientation improved significantly with age, without other sources of error changing.

## 4 Discussion

The current study investigated longitudinal development of VWM precision during childhood and early adolescence. Forty participants aged 7–11 years at *t*1 completed a VWM precision task battery twice, two years apart. Results demonstrate firstly that recall precision increased with age on both 1-item and 3-item sequential VWM precision tasks. These increases remained significant after controlling for age-related improvement in performance on a sensorimotor control task. Second, the longitudinal effects of age were attributable to a specific decrease in variability in the representation of target stimuli, and not due to changes in random responding or to misbinding, i.e. corruption by features of other items retained in memory. We discuss implications in terms of developmental mechanisms underlying observed improvements in VWM performance with age.

### 4.1 Development of working memory precision

A number of studies have shown improvement across childhood in performance on standard tests of VWM (Alloway et al., 2006, Gathercole et al., 2004). In the current study, we contribute to understanding the mechanistic underpinnings of VWM development. We show that the precision with which items are recalled from VWM, whether presented individually or in sequences of three, increased after a time interval of two years in participants aged 7–11 years at study entry. Because participants were sampled longitudinally, we can rule out interpretations based wholly or partially on inter-individual variability and cohort effects, enabling conclusions to be drawn regarding the developmental trajectory of VWM precision. Importantly, longitudinal increases in precision withstood correction for improvements in performance on a control task requiring fine hand-eye co-ordination, and hence are not readily explicable on the basis of improvement in sensorimotor factors. Instead, the results suggest that the resolution of items recalled from VWM increases during middle childhood and early adolescence.

These conclusions align with those from cross-sectional studies showing age-associated development during childhood and adolescence in VWM performance (Burnett Heyes et al., 2012, De Luca et al., 2003; Luciana, Conklin, Hooper, & Yarger, 2005; Swanson, 1999, Zald and Iacono, 1998). The current results are also consistent with studies showing developmental improvements in performance on executive tasks that have a VWM component (Brocki and Bohlin, 2004, Luciana et al., 2005; Luna, Garver, Urban, Lazar, & Sweeney, 2004). Using continuous recall measures, rather than discrete measures of capacity, may offer enhanced sensitivity for tracking behavioural changes in VWM, as has recently been demonstrated using even relatively small samples (N = 12) of adult patients with neurodegenerative conditions (Zokaei et al., 2014). Importantly, the approach taken here also enables investigation of the underlying cognitive mechanisms associated with these developmental changes.

### 4.2 Modelling the distribution of responses

Continuous VWM tasks provide a means to examine the sources of error in recall. We applied a probabilistic model (Bays et al., 2009) to response data from the 3-item VWM task to decompose the contributions of different sources of error. Within this model, error in memory can arise from three sources. Firstly, it can be due to variability – ‘noise’ – in memory for the remembered feature. Alternatively, error can arise due to random guessing, for example due to failures at encoding or retrieval. Lastly, response error in the 3-item VWM task may also theoretically arise due to systematic interference or biasing of information by features belonging to other items encoded into VWM (i.e. misbinding or non-target responses). Here, we show that improvement with age in the 3-item VWM task was attributable to a specific decrease in variability in the representation of target stimuli, and could not be attributed to any changes in the frequency of guesses or misbinding errors. As such, our modelling analysis sheds new light on potential specific mechanisms underlying longitudinal development in VWM performance.

In future, delayed reproduction VWM tasks accompanied by probabilistic modelling of response data could also provide a useful tool for characterising more fully neural changes associated with VWM across the lifespan. Several studies have shown that it is possible to track development of the functional neural substrates of VWM during childhood and adolescence (Bunge and Wright, 2007, Dumontheil and Klingberg, 2012; Geier, Garver, Terwilliger, & Luna, 2009; Klingberg, Forssberg, & Westerberg, 2002). Using precision, rather than traditional measures of capacity, may offer enhanced sensitivity for detecting variance associated with age.

### 4.3 Differential development on 1-item and 3-item VWM tasks

The current study showed effects of age on the *difference* in recall precision between 1-item and 3-item tasks, conceptually equivalent to an age by task interaction. That is, whereas we demonstrate significant improvement with age on both VWM tasks, improvement on the harder 3-item task was more substantial. Further empirical studies are needed to explore the developmental cognitive mechanisms underlying this result. Possibilities include development of attention (Astle, Nobre, & Scerif, 2012) or even emerging metacognitive capability (Weil et al., 2013).

## 5 Conclusion

We present longitudinal evidence that the *precision* of VWM for individually and multiply-encoded items develops throughout middle childhood and early adolescence. This development is attributable to a specific decrease in variability – noisiness – of stored feature representations. This highlights a mechanism that may underlie longitudinal improvement in VWM performance, without invoking improvement in the number of items that can be stored.

These findings demonstrate protracted development in VWM performance and shed light on the potential mechanisms which could underpin this development longitudinally. Currently, there is much interest in modifying cognition as a means of addressing suboptimal developmental trajectories (Klingberg et al., 2005). VWM recall precision might provide a sensitive metric for assessing the impact of behavioural training and neurofeedback interventions (Wass, Scerif, & Johnson, 2012).

## Figures and Tables

**Table 1:** Participant characteristics at time 1 and time 2.

	Age (N = 40)		FSIQ^e^ (N = 40)	
	Mean (SD)	Range	Mean (SD)	Range
*t*1	10.2 (1.02)	7.9–11.7	111 (13.1)	81.5–133
*t*2	12.2 (1.02)	9.9–13.7	–	–

**Table 2:** Mean (SD) precision values (rad^−1^) in each task by time-point and condition. All VWM precision values are corrected for sensorimotor precision. One outlying data point was excluded (see text).

	Sensorimotor	1-item VWM	3-item VWM			
			Total	SP 1	SP 2	SP 3
*t*1 (N = 40)	8.74 (2.73)	3.33 (1.43)	2.33 (1.08)	1.90 (1.15)	1.83 (.496)	3.26 (2.29)
*t*2 (N = 40)	10.30 (2.42)	4.25 (1.69)	2.80 (1.19)	2.24 (1.31)	2.16 (0.63)	4.01 (2.67)
